# Emerging Biomaterials for Maxillary Sinus Augmentation: From In Vitro Insights to In Vivo Clinical Translation

**DOI:** 10.3390/ma19040737

**Published:** 2026-02-14

**Authors:** Nicole Riberti, Michele Furlani, Alessandra Giuliani

**Affiliations:** 1 Department of Clinical and Molecular Sciences, Marche Polytechnic University, 60126 Ancona, Italy; n.riberti@staff.univpm.it; 2 Department of Biomedical Sciences and Public Health, Marche Polytechnic University, 60126 Ancona, Italy; 3 Department of Clinical Specialistic and Dental Sciences, Marche Polytechnic University, 60126 Ancona, Italy

**Keywords:** maxillary sinus augmentation, biomaterials, deproteinized bovine bone mineral, bone regeneration, translational research

## Abstract

Maxillary sinus augmentation is a key procedure for rehabilitating the atrophic posterior maxilla and enabling predictable implant-supported restorations. Although autogenous bone remains the biological gold standard due to its osteogenic potential, its clinical use has declined because of donor-site morbidity, limited availability, and increased surgical burden. Deproteinized bovine bone mineral (DBBM) is currently the most widely used substitute, providing excellent biocompatibility and long-term volumetric stability. However, its inert nature, limited bioactivity, and slow resorption have driven the development of next-generation graft materials. Recent biomaterial innovations aim to enhance vascularization, accelerate osteogenesis, modulate immune responses, and achieve controlled resorption while maintaining favorable handling properties. These include ion-releasing bioactive ceramics, growth factor-enhanced allografts, polysaccharide–hydroxyapatite composites, smart hydrogels, and synthetic scaffolds with tunable degradation profiles. Given the complexity of bone regeneration, effective clinical translation requires an integrated framework combining in vitro assays, animal models, and human clinical studies. This review synthesizes evidence published since 2018 on emerging biomaterials for sinus floor elevation, critically evaluating their potential to overcome the limitations of DBBM and highlighting the importance of a coordinated preclinical-to-clinical research continuum.

## 1. Introduction

Maxillary sinus augmentation is a well-established and widely adopted surgical intervention for managing the atrophic posterior maxilla and enabling predictable implant-supported rehabilitation. Among the traditional grafting materials available—autogenous bone, xenogeneic bone, demineralized or mineralized allogeneic bone, and alloplasts [1]—autogenous bone has traditionally been regarded as the gold standard due to its unique combination of osteogenic, osteoinductive, and osteoconductive properties [2]. Its inherent biological activity supports rapid graft integration and the formation of vital, mature bone, outperforming all currently available biomaterials. Nevertheless, the clinical use of autogenous grafts has progressively declined, largely because procurement requires a secondary surgical site, prolongs operative time, and is associated with donor-site morbidity, postoperative discomfort, and limited graft volume [3]. Younger and Chapman [4] reviewed 243 autogenous bone graft procedures and reported an overall major complication rate of 8.6%. Major complications included infection, prolonged wound drainage, reoperation, pain persisting for more than six months, and sensory loss. Minor complications—defined as superficial infection, minor wound-related issues, temporary sensory disturbances, and mild or resolving pain—were observed in 20.6% of patients. The morbidity associated with autologous bone harvesting was found to be strongly influenced by the choice of the donor site. In particular, Younger and Chapman also reported a substantially higher major complication rate (17.9%) when the surgical incision was also used for graft harvesting [4]. The morbidity related to iliac crest harvesting has been well documented and includes bleeding, fracture, neurological injury, and significant postoperative pain. Evidence suggests that the posterior iliac crest may be associated with fewer complications than the anterior crest [5]. Nevertheless, donor-site morbidity appears to be reduced when smaller graft volumes are harvested. In this regard, for instance, Suda et al. reported only minor complications following the use of iliac crest bone grafts for the treatment of distal radius fractures [6].

However, these drawbacks have contributed to a pronounced shift toward xenogeneic substitutes, particularly deproteinized bovine bone mineral (DBBM). DBBM has become the most widely used grafting material in sinus floor elevation, supported by extensive preclinical and clinical evidence demonstrating excellent biocompatibility and long-term volumetric stability [7,8]. Its highly crystalline, slowly resorbing structure ensures sustained space maintenance, facilitating predictable implant placement. However, despite its widespread clinical success, DBBM functions primarily as a passive osteoconductive scaffold and lacks intrinsic osteogenic or angiogenic activity. Its limited biodegradation may also result in persistent non-vital particles, potentially constraining the quantity and biological quality of the newly formed bone.

Growing insights into bone biology, coupled with the need for biomaterials that are biologically active, ethically acceptable, and capable of supporting faster and more predictable regeneration, have stimulated the development of innovative grafting alternatives [9]. These emerging materials seek to address the limitations of DBBM by enhancing early vascularization, stimulating osteogenesis, improving handling characteristics, achieving complete and timely resorption, and interacting more dynamically with the host immune and regenerative environment [10]. In particular, adequate vascularization is critically important in the maxillary sinus environment for several reasons related to surgical success, tissue regeneration, wound healing, and the prevention of complications, especially in commonly performed procedures such as sinus floor augmentation and dental implant placement. Current research avenues include ion-releasing bioactive ceramics, growth factor–enriched or autologous-derivative allografts, hybrid polysaccharide–hydroxyapatite composites, smart hydrogels capable of binding regenerative molecules, and synthetic scaffolds with tunable degradation kinetics and bioactivity. These emerging biomaterials are also poised to impact clinical decision-making by offering enhanced predictability, reducing procedural complexity, and minimizing patient morbidity compared to traditional methods [11,12].

As these biomaterials advance through early stages of development, the translational pathway from laboratory testing to clinical implementation becomes a central consideration. Robust evaluation requires coordinated investigation across in vitro, animal, and human models, each offering complementary insights. In vitro studies provide rapid, cost-effective, and ethically sound information on cytocompatibility, cell adhesion, osteoblastic activity, and material stability, although they cannot replicate the complexity of vascularization, immune interactions, mechanical loading, or the sequential events of bone healing [13]. Animal models, therefore, remain indispensable in preclinical research, offering biologically integrated systems in which biomaterials interact with vascular, immune, and skeletal tissues under physiological conditions. Small animals enable high-throughput assessment of early healing mechanisms, while large animals more closely replicate human sinus anatomy and bone remodeling dynamics. Yet, interspecies differences in anatomy and physiology necessitate caution when extrapolating results to humans [14].

Ultimately, human in vivo studies provide the highest level of evidence for the long-term performance, functional stability, and clinical relevance of new grafting materials. Although they cannot deliver the mechanistic resolution afforded by in vitro or animal studies, clinical trials confirm whether preclinical findings translate into predictable outcomes under real-world conditions.

A modern framework for biomaterial evaluation, therefore, depends on an integrative, multi-level research continuum: (i) in vitro studies for preliminary mechanistic and cytocompatibility screening; (ii) animal models for assessing biological integration, regenerative potential, and safety; and (iii) in vivo human investigations for functional validation and long-term clinical success.

**Figure 1:**
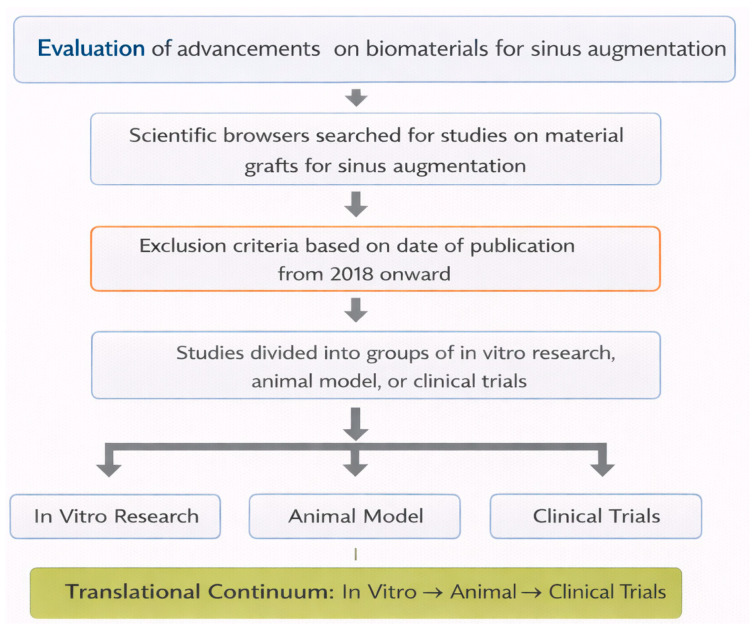
Logical workflow and aim of the present work. The choice of selecting papers from 2018 onward complements the comprehensive review by Al-Moraissi et al. [15].

The present review synthesizes (Figure 1) evidence from 2018 to the present regarding emerging biomaterials for sinus floor elevation, examining their biological and clinical performance across in vitro, animal, and human models. Particular emphasis is placed on how these materials address the limitations of DBBM and on the importance of a coordinated translational pathway for the safe and effective introduction of novel graft substitutes. Indeed, maxillary sinus augmentation remains a cornerstone procedure in implant dentistry, and the continuous introduction of emerging biomaterials onto the market renders this review both timely and clinically relevant for researchers and practitioners. To address this evolving landscape, we adopted a structured translational framework that establishes a logical parallel between experimental evidence and clinical application, guiding the reader from in vitro investigations to in vivo and clinical studies published from 2018 onward. This temporal cutoff was selected because the previous review by Al-Moraissi et al. [15] had already provided a comprehensive and authoritative overview of the state of the art up to that time. The translational approach employed herein was intended to strengthen the scientific narrative and enhance the practical relevance of the review.

## 2. In Vitro Studies

In vitro research provides the foundational mechanistic insight required to evaluate and optimize emerging biomaterials for maxillary sinus augmentation. These models enable controlled assessment of cytocompatibility, early osteogenic responses, and physicochemical stability before advancing to preclinical animal testing. Among the most commonly applied methodologies are assays of cell viability and adhesion, alkaline phosphatase (ALP) activity, ion-release profiling, simulated body fluid (SBF) mineralization tests, and comprehensive surface chemistry and material characterization analyses. Collectively, these approaches allow researchers to determine how variations in composition, fabrication techniques, and surface treatments influence cell behavior and early osteogenic signaling.

**Figure 2:**
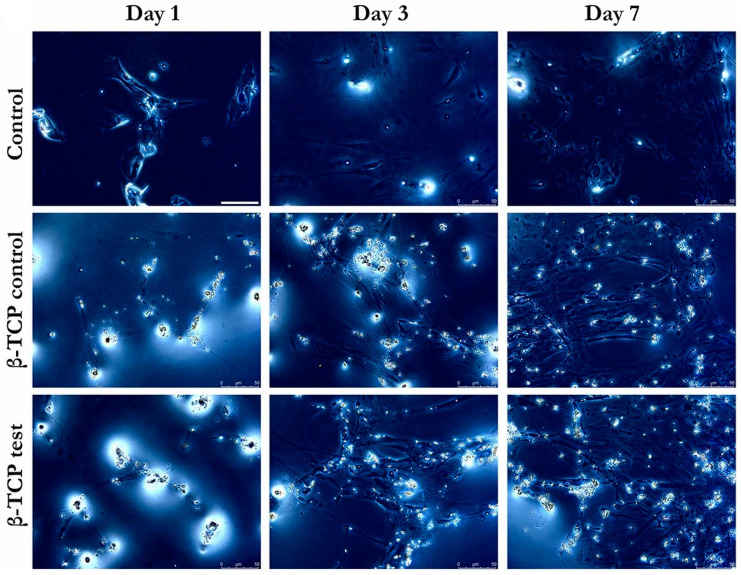
Morphological changes in human mesenchymal stem cells on argon glow–discharge plasma-treated β-TCP at days 1, 3, and 7. Day 1: Sparse spindle-shaped cells observed across all media. Day 3: Cells appeared more spread and showed increased proliferation. Day 7: Cells exhibited an advanced developmental stage with evident filopodial extensions. Magnification: 40×. Adapted from Ref. [16].

Recent investigations have explored a variety of strategies aimed at enhancing the biological performance of graft substitutes, reflecting a broader shift in biomaterials science from the development of passive osteoconductive scaffolds toward the design of materials capable of actively modulating the regenerative microenvironment [9]. One such example is argon glow–discharge plasma-treated β-tricalcium phosphate (β-TCP), which represents a minimalist yet effective refinement [16]. By removing micro-debris without altering the bulk morphology or topography of the material, this treatment significantly improves early cell adhesion and osteogenic gene expression, illustrating how surface purity and chemistry alone can shape cellular fate, as illustrated in Figure 2.

In contrast to this subtle modification, the development of Matrix-HA exemplifies a composite-based engineering strategy. This injectable system integrates natural polysaccharides with hydroxyapatite (HA) to create bioactive microbeads capable of supporting endothelial cell proliferation and promoting early angiogenesis—an essential feature for grafts placed in large, poorly vascularized environments such as the maxillary sinus [17]. This approach emphasizes the importance of coupling osteoconduction with vascular maturation to achieve predictable bone regeneration.

The Blue Bone nanocomposite advances the field further by leveraging nanoscale engineering. Produced through highly controlled manufacturing processes, this hydroxyapatite/β-tricalcium phosphate (HA/β-TCP) nanocomposite exhibits high porosity and an enlarged surface area, characteristics that closely emulate the architecture of native bone mineral. These features enhance cytocompatibility, promote early vascular integration, and support the initial phases of osteogenesis, highlighting the potential of nanostructured surfaces to orchestrate complex regenerative processes [18].

Finally, magnesium-enriched β-TCP illustrates the growing interest in chemical biofunctionalization. Incorporating MgO into β-TCP without modifying its surface morphology enables the material to stimulate osteoblastic differentiation and augment early bone formation. This underscores the physiological significance of ion-mediated signaling pathways in bone metabolism and demonstrates how targeted chemical doping can confer bioactivity beyond that of traditional calcium phosphate ceramics [19]. Indeed, modest increases in Mg^2+^ availability within ranges known to be physiologically relevant can activate osteogenic signaling pathways and enhance early osteoblastic differentiation. In vivo, these effects are further modulated by local blood flow, adsorption to the biomaterial surface, incorporation into the mineralizing matrix, and systemic homeostatic regulation [20].

Taken together, these studies illustrate a clear and progressive evolution in biomaterial design. Innovations range from surface purification strategies to hybrid composite systems, to nano-engineered architectures, and finally to ion-sensitive biofunctionalization. Each approach provides a distinct mechanistic contribution toward creating graft materials that are not only structurally supportive but also biologically interactive, ultimately aiming to improve the predictability and quality of bone regeneration in sinus augmentation procedures.

## 3. Animal Models

### 3.1. Deproteinized Bovine Bone Materials

A substantial proportion of the available preclinical literature continues to focus on DBBMs, reflecting its long-standing prominence in sinus augmentation research. Across multiple rabbit and mouse models, DBBM consistently maintains graft volume but exhibits characteristic biological behavior. For example, studies examining different DBBM particle sizes in rabbits demonstrated that the Schneiderian membrane progressively thinned over time regardless of granule dimensions, with perforations becoming increasingly common by eight weeks—suggesting that this response is intrinsic to the material rather than size-dependent [21]. This observation supports the continued emphasis on meticulous surgical technique and conservative membrane handling in DBBM-based human studies.

The incorporation of collagen into DBBM produced more nuanced outcomes [22]. During the early healing phase (two weeks), collagenated DBBM showed a transient protective effect on the sinus membrane, with fewer thinning regions compared to standard DBBM. However, by twelve weeks, this advantage dissipated, and both materials displayed similar degrees of membrane thinning and perforation. Bone formation remained comparable between the two formulations. These findings are considered a rationale for why collagenated formulations have not consistently translated into superior clinical outcomes and why later-stage clinical trials have focused on equivalence rather than superiority endpoints.

Efforts to biologically enhance DBBM through supplementation with hyaluronic acid or polynucleotides did not yield measurable improvements [23]. In a rabbit model, these adjuncts failed to accelerate early bone regeneration or mitigate membrane complications relative to DBBM alone, indicating that the intrinsic biological limitations of DBBM are not easily overcome by simple additives. Thus, simple biological supplementation strategies have not been prioritized in clinical trial pipelines.

Additional evidence from a mouse model comparing autograft with DBBM further highlights these limitations [24]. Autografts demonstrated rapid early bone formation and strong alkaline phosphatase activity, whereas DBBM elicited a delayed osteogenic response. By later time points (approximately day 18), both materials supported new bone.

Collectively, these findings reinforce that DBBM is a reliable and volumetrically stable graft, yet biologically passive and slower to integrate during the early stages of healing.

### 3.2. Porcine-Derived Xenografts

Porcine-derived grafts represent a relevant alternative to bovine xenografts, and several preclinical studies have evaluated their performance in sinus augmentation models. In one investigation, a “sticky” dual-phase porcine substitute was compared with a conventional particulate porcine graft in rabbits [25]. Despite theoretical advantages related to cohesiveness and ease of handling, the sticky formulation did not demonstrate superior biological outcomes. Both materials exhibited substantial volume reduction—approximately 50% by ten weeks—yet maintained graft integrity even in the presence of experimentally induced Schneiderian membrane perforations.

**Figure 3:**
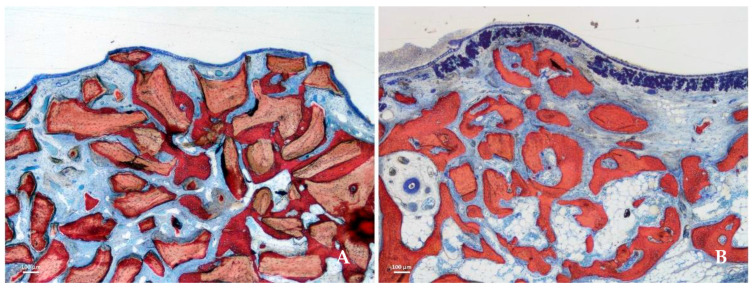
Photomicrograph of ground section after 8 weeks of healing. Higher soft tissue content was present in the collagenated group (**A**) than in the DBBM group (**B**), and the difference was statistically significant in all regions (*p* < 0.001). Stevenel’s blue and alizarin red stain were used. Reprinted from Ref. [26].

A separate rabbit study directly compared a collagenated porcine xenograft with bovine-derived DBBM [26]. The porcine material showed faster degradation and greater shrinkage of the augmented space; however, it also induced increased bone formation along the sinus walls (Figure 3). These findings suggest that porcine xenografts may promote more active remodeling dynamics, albeit at the expense of long-term volumetric stability.

Further insights are provided by a beagle dog study assessing particulate porcine bone versus a collagenated porcine block [27]. The block configuration enabled quicker graft placement, offering a practical advantage, yet both formats yielded comparable amounts of new bone, residual graft, and osseointegration after eight weeks. Overall, porcine xenografts appear to provide regenerative outcomes similar to DBBM, while differing primarily in their resorption behavior and handling properties.

These findings help explain why clinical adoption of such formulations has been driven primarily by surgical convenience rather than expectations of superior bone regeneration.

### 3.3. Synthetic Polymers and Composite Materials

A diverse range of synthetic and composite biomaterials has been explored in recent years, reflecting the growing interest in developing graft substitutes that are fully resorbable, angiogenic, and biologically active. In sheep, poly(lactic-co-glycolic acid) (PLGA)-based scaffolds demonstrated encouraging performance over mid-term healing periods (16–28 weeks), exhibiting substantial bone remodeling and integration without adverse tissue reactions. Although limited by small sample sizes, these findings suggest that PLGA may support predictable long-term bone maturation [28]. These PLGA-based scaffolds, while not intended for load-bearing applications, provide sufficient initial structural integrity to maintain defect volume during early healing, with gradual load transfer to newly formed bone as degradation progresses. Their tunable degradation kinetics allow a balance between temporary mechanical support and timely resorption [29]. This biological rationale supports their consideration for clinical scenarios requiring fully resorbable, temporizing scaffolds rather than permanent space maintainers.

In contrast, the vitroceramic material Biosilicate^®^ produced mixed outcomes in rabbit models. While bone formation did occur, persistent inflammation and granulation tissue were consistently reported, raising concerns regarding its long-term biocompatibility for sinus applications [30]. These preclinical findings are explicitly cited as reasons why such materials have not progressed to clinical sinus augmentation studies, underscoring the role of animal models in de-risking material selection before human application.

A more engineering-oriented strategy has led to the development of self-healing gelatin/nanoclay colloidal gels that are both injectable and 3D-printable, offering tunable viscoelasticity and high elastic moduli. Evaluated in small-animal sinus augmentation and critical-size cranial defect models, these gels supported cell adhesion and osteogenic differentiation in vitro, and facilitated bone ingrowth in vivo, with the scaffolds undergoing gradual biodegradation and replacement by new bone. Their key advantages include minimally invasive delivery, excellent conformity to irregular defects, and the potential for patient-specific fabrication. Short-term animal studies indicate favorable osteointegration and resorption behavior, positioning them as promising candidates where handling precision is clinically important [31]. For these specific biomaterials, mechanical stability is primarily relevant in terms of viscoelasticity, resistance to collapse, and shape fidelity rather than classical load-bearing capacity. These materials are designed to conform to irregular defects and maintain space under low mechanical stresses, while their relatively high elastic moduli and self-healing properties support handling precision and stability during the early regenerative phase [31].

Porous titanium granules represent an alternative strategy, functioning as a non-resorbable yet highly osteoconductive scaffold. In sheep models, they induced significantly more new bone formation than empty controls and exhibited strong integration. However, their permanent nature means they remain indefinitely in situ, a characteristic that may be advantageous or limiting depending on clinical objectives [32]. Porous titanium granules provide excellent long-term mechanical stability and resistance to deformation, acting as a permanent osteoconductive framework. While this characteristic ensures sustained space maintenance, their non-resorbable nature may limit physiological bone remodeling and adaptability over time, making their mechanical permanence a double-edged feature depending on clinical objectives [33].

### 3.4. Growth-Factor-Enhanced Biomaterials

A small but noteworthy subset of studies has investigated biomaterials augmented with osteogenic growth factors.

In mini-pig models, recombinant human bone morphogenetic protein-2 (rhBMP-2) delivered on an absorbable collagen sponge produced pronounced osteoinductive effects, and the addition of a biphasic ceramic scaffold failed to provide further enhancement. This indicates that the potent biological activity of bone morphogenetic protein-2 (BMP-2) largely dictates the regenerative response, irrespective of the underlying scaffold composition [34].

Similarly, in rat models, an injectable heparin-modified gelatin nanofibrous scaffold engineered for sustained BMP-2 release generated the highest levels of osteogenesis among all tested materials. These findings underscore the substantial regenerative capacity of growth-factor-enhanced scaffolds, while also emphasizing that their clinical translation requires rigorous evaluation of dosage, delivery control, safety, and regulatory constraints [35].

Table 1 provides a concise summary of the four principal avenues for biomaterial innovation in maxillary sinus floor augmentation.

## 4. Clinical Cases

Across the collected clinical studies, several distinct research intentions emerge, each reflecting a different way in which biomaterials and biologically active adjuncts are being used to advance sinus floor augmentation beyond traditional xenograft-based techniques. Table 2 synthesizes the intent and the main results of each study, highlighting the biomaterial under examination.

One group of studies focuses on fully synthetic alternatives, aiming to replace or enhance the performance of deproteinized bovine bone, which—despite its long-term clinical success—remains biologically inert and slow to remodel. The octacalcium phosphate (OCP)-based synthetic granules [36] and the Mg-hydroxyapatite/collagen hybrid scaffold [37] represent two examples of this direction. Both materials are designed to be actively resorbable and biologically instructive, with OCP serving as a precursor to biological apatite and Mg-enriched HA encouraging osteoblast differentiation and vascularization. Their clinical biopsies showed robust early bone formation and, in the case of the magnesium–collagen hybrid, complete scaffold disappearance by six months, suggesting that next-generation synthetics may be capable of achieving the same volumetric stability as xenografts but with far greater biological turnover.

A second set of studies explores biologically enhanced grafts, where the goal is not only to fill space but to accelerate regeneration through growth factors, autologous concentrates, or bioactive coatings. Platelet-Rich Fibrin (PRF), whether used alone or combined with allografts or xenografts, is repeatedly shown to stimulate early vascularization, increase bone–material contact, and potentially shorten healing time. In some cases, PRF alone achieved sinus augmentation height gains comparable to allografts, highlighting the possibility of graftless, biology-driven approaches [38,39]. Similarly, studies using albumin-coated allografts [40] or BMP-2 in human biopsies report substantial gains in early osteogenesis and bone quality, emphasizing that cellular and molecular enhancement may be as crucial as the material scaffold itself. These approaches underscore a paradigm shift: the graft is no longer merely a passive filler but becomes a bioactive initiator of healing.

A third major theme involves comparative clinical evaluations of existing graft materials, reflecting the need to refine or replace long-standing standards like Bio-Oss^®^. The randomized trial comparing six widely used biomaterials [41], ranging from bovine xenografts to mineralized allografts and synthetic HA/β-TCP, demonstrated that all materials were clinically viable at six months, but allografts (particularly mineralized Freeze-Dried Bone Allograft) produced the highest new bone fraction. Likewise, comparing Cerabone^®^ and Bio-Oss^®^ revealed significant differences in bone regeneration despite both being DBBM derivatives [42]; Cerabone^®^ resulted in a greater proportion of vital bone, challenging the notion that all xenografts behave equivalently. These findings suggest that clinical outcomes depend heavily on subtle differences in processing, mineral crystallinity, and material purity, highlighting the need for more granular evaluation rather than grouping grafts solely by origin. Meanwhile, another study examining the impact of placing or omitting a collagen membrane over the lateral window [43] demonstrated no difference in bone regeneration, suggesting that this common step may be unnecessary in many sinus augmentation procedures.

**Figure 4:**
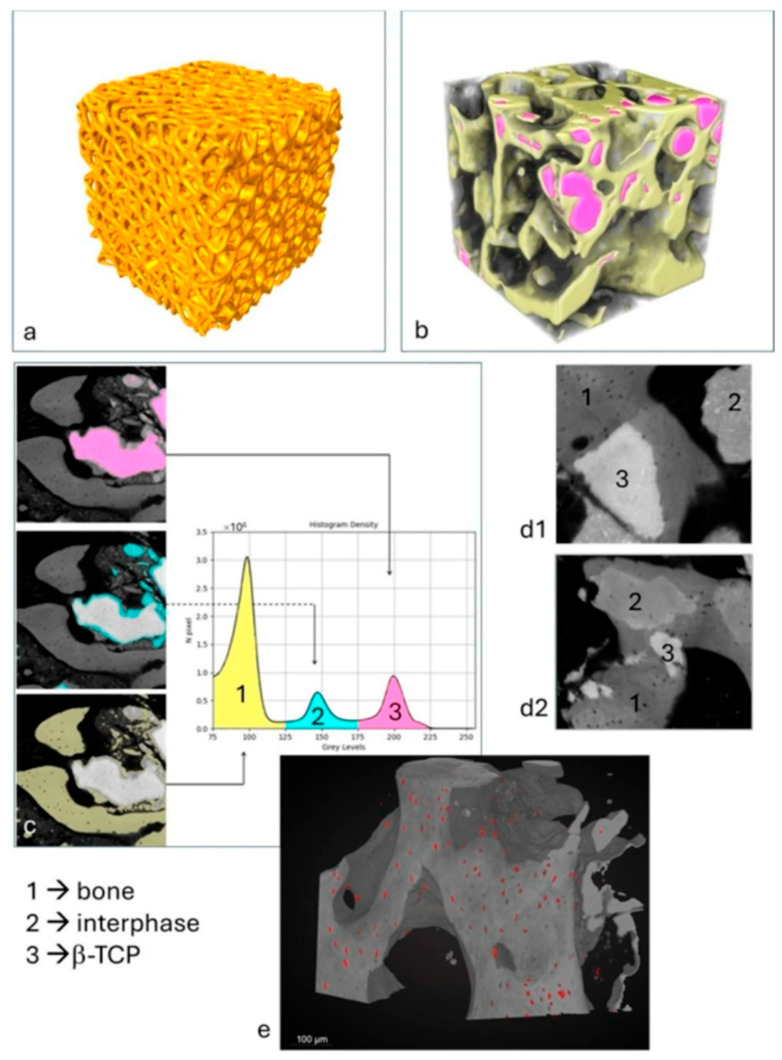
Micro-CT and Phase-Contrast micro-CT imaging analysis: (**a**) Microarchitecture of the scaffold shown in a representative sample. (**b**) micro-CT reconstruction of a sub-volume from the patient’s biopsy: yellow = newly formed bone; pink = residual β-TCP. (**c**) Density histogram from a representative biopsy region: peak 1 = newly formed bone; peak 2 = interphase; peak 3 = residual β-TCP. (**d1**,**d2**) Additional biopsy regions imaged with Phase-Contrast micro-CT, illustrating biomaterial integration and resorption. (**e**) Representative sub-volume of the biopsy showing osteocyte lacunae segmented by the neural network, displayed in red. Reprinted from Ref. [45].

Finally, several studies introduce innovations not only through the development of novel graft materials, but also by refining procedural workflows and advancing the analytical tools used to investigate graft remodeling. The investigation that used biphasic calcium phosphate scaffolds as a model to track the temporal evolution of mineral and collagen structures [44] illustrates how advanced spectroscopic and microstructural analyses can substantially deepen our understanding of graft maturation (Figure 4). Similarly, lithography-based β-TCP scaffolds were analyzed with respect to their crystalline structure, microarchitecture, mechanical behavior, and biological performance, and this characterization was complemented by an in vivo human biopsy obtained six months after sinus augmentation. Histology, X-ray micro-CT, synchrotron imaging, and Fourier Transform Infrared (FTIR) analyses confirmed substantial new bone formation, the presence of organized osteocyte lacunar networks, and progressive β-TCP dissolution. Taken together, these findings validate the scaffold as a biocompatible, osteoconductive, and structurally optimized biomaterial suitable for sinus floor elevation and complex jawbone reconstruction [45]. The overview of autogenous versus alternative grafts in sinus augmentation [46] helps contextualize these findings by emphasizing the clinical need for predictable, minimally invasive regeneration in an anatomically challenging region. These studies indicate that progress in sinus augmentation is not limited to material innovation alone; advances in diagnostic imaging, biomechanical modeling, and surgical planning are equally essential for improving clinical outcomes.

## 5. Discussion

Research on biomaterials for maxillary sinus augmentation has progressed from an early dependence on passive osteoconductive xenografts to a more sophisticated understanding of how material chemistry, architecture, and biological functionalization influence the regenerative microenvironment. Evidence from in vitro experiments, animal models, and human clinical studies outlines a consistent trajectory: contemporary grafts are being engineered not simply to occupy space, but to modulate osteogenesis, vascularization, and controlled remodeling.

In vitro investigations provide the first layer of insight by isolating cell responses to precisely tuned modifications in biomaterial composition and surface characteristics. Multiple studies show that even subtle physicochemical changes significantly enhance cell–material interactions. Examples include the removal of micro-debris from β-TCP through glow-discharge plasma, magnesium-doped β-TCP, nano-engineered HA/β-TCP composites (e.g., Blue Bone), and more sophisticated systems such as polysaccharide–HA microbeads or gelatin/nanoclay colloidal gels. Collectively, these findings demonstrate that nano- and micro-scale innovations can meaningfully reshape the biological behavior of otherwise conventional calcium-phosphate scaffolds, providing a predictive foundation for their in vivo performance. However, current in vitro models used to investigate biomaterials for maxillary sinus augmentation present inherent limitations, as they often rely on simplified monoculture systems that do not fully reproduce the complex biological environment of the maxillary sinus [47,48]. In particular, the absence of vascular components, immune cells, and the dynamic interactions between different cell populations limit their ability to predict in vivo behavior and clinical outcomes. Moreover, commonly used cell types, such as osteoblast-like cells or mesenchymal stem cells, fail to capture the specific contributions of sinus membrane–derived cells and endothelial cells to angiogenesis and tissue regeneration [49,50].

Moreover, in vitro findings should be interpreted as mechanistic and screening tools that inform material optimization and guide preclinical study design, rather than as direct predictors of long-term clinical outcomes. Indeed, early in vitro outcomes, such as cell adhesion, viability, alkaline phosphatase activity, and early osteogenic gene expression, do not directly predict long-term bone formation; however, they provide critical surrogate markers of a biomaterial’s initial biological performance. These early events are closely linked to subsequent in vivo processes, as initial cell–material interactions, surface-driven protein adsorption, and early osteogenic signaling influence angiogenesis, matrix deposition, and the progression toward mature bone formation [51,52]. In particular, in the context of maxillary sinus augmentation, favorable early in vitro responses are generally associated with improved early host integration, reduced inflammatory reactions, and more predictable osteoconductive behavior in vivo [53]. While long-term graft performance is ultimately determined by complex biological and mechanical factors that cannot be fully replicated in vitro, materials that demonstrate enhanced early osteogenic and surface-mediated responses tend to show more consistent bone formation and remodeling in preclinical and clinical studies. This clarification helps to position early in vitro data within a translational continuum that progresses from surface–cell interactions to in vivo bone regeneration and long-term graft performance.

Animal studies add a second layer of evidence by placing these materials within the mechano-biologically complex environment of the sinus cavity. Across rabbit, sheep, pig, and rodent models, DBBM consistently maintains volume yet exhibits predictable limitations, including slow turnover, minimal bioactivity, and progressive Schneiderian membrane thinning. Attempts to enhance DBBM with collagen or bioactive gels improved early healing but did not overcome its intrinsic biological inertia. By contrast, porcine xenografts showed more rapid remodeling and greater tissue dynamics, although with reduced long-term stability. Synthetic options—such as PLGA-based constructs, polysaccharide–HA matrices, and colloidal gels—often integrated well, sometimes exceeding xenografts in vascularization and early bone formation, especially in large-animal models approximating human sinus anatomy. Scaffolds enriched with growth factors, particularly BMP-2, consistently elicited robust osteoinductive responses, emphasizing the therapeutic potential of biologically activated materials irrespective of scaffold type. Overall, animal studies reveal a diverse spectrum of grafts under evaluation: DBBM remains the standard for structural stability despite limited biological activity; porcine xenografts offer comparable regenerative outcomes with distinct resorption patterns; synthetic composites—especially polysaccharide–HA systems—show promise in balancing bioactivity, biocompatibility, and degradation; and growth-factor-loaded scaffolds remain the most potent stimulators of bone formation, though their clinical use requires stringent oversight.

Notably, repeated observations of Schneiderian membrane thinning and occasional perforation associated with DBBM in preclinical studies have prompted concerns regarding its biological behavior at the sinus–membrane interface [54]. In animal models, these findings are often attributed to the persistence and slow resorption of DBBM particles, which may exert prolonged mechanical contact and induce localized remodeling or adaptive thinning of the membrane. However, DBBM-related membrane alterations—observed in animal models—appear limited and context dependent if referred to the clinical impact in human sinus augmentation. Indeed, available human data suggest that DBBM is generally well tolerated in sinus augmentation procedures, with a low incidence of clinically relevant membrane complications when appropriate surgical techniques are employed [55]. Importantly, membrane thinning observed in experimental settings does not necessarily translate into clinically significant perforations in humans, likely due to differences in membrane thickness, healing capacity, surgical handling, and postoperative loading conditions. Moreover, in clinical practice, Schneiderian membrane perforation is more commonly related to intraoperative mechanical factors rather than to the intrinsic properties of the graft material [56]. Overall, DBBM’s “biological passivity” represents a trade-off rather than a limitation per se, and highlights the need for indication-driven material selection that balances regenerative kinetics, volumetric demands, and clinical predictability.

Indeed, human clinical research completes the evidence framework by assessing safety, predictability, and translational relevance. New-generation synthetics such as octacalcium phosphate or magnesium-substituted HA/collagen hybrids demonstrate that fully resorbable, bioactive substitutes can achieve bone volumes and quality comparable to xenografts within clinically acceptable healing intervals. Multiple randomized and controlled trials indicate that several allografts and BCP-based synthetics produce greater amounts of new bone at six months than DBBM, thereby challenging longstanding assumptions about xenograft superiority. Concurrently, biologically enhanced approaches—including PRF-enriched grafts and albumin-coated allografts—appear capable of shortening healing time and improving early vascularization, a key determinant of sinus augmentation success. Additional studies focused on workflow optimization and monitoring technologies, with MRI and synchrotron imaging enabling high-resolution, radiation-free assessment of graft maturation over extended periods. Taken together, clinical findings show that sinus augmentation is advancing along several complementary pathways. Synthetic and ion-modified materials aim to replicate or outperform xenografts through controlled resorption and favorable biological signaling; biologically enriched grafts seek to accelerate healing by amplifying endogenous regenerative cascades; comparative trials refine our understanding of graft performance under standardized conditions; and innovations in imaging and scaffold fabrication reflect a growing emphasis on procedural precision and patient-specific solutions. The overall trajectory suggests that future progress in sinus augmentation will derive not from a single superior material but from strategic combinations of biomimetic scaffolds, biologically active cues, and digitally supported surgical innovations. The wide use of new polymeric matrices enhanced with ceramic materials or xenografts supports this hypothesis, and to better understand how impactful each of the components is in this new scenario, Table 3 reports a schematic overview of the main aspects and reasons the new research points in this direction.

Across all levels of evidence, several converging themes emerge. DBBM remains a dependable space-maintaining scaffold yet is biologically passive, prompting the design of newer materials that address this limitation. Synthetic and composite grafts now rival or surpass xenografts in early bone formation, particularly those incorporating bioactive ions, nano-structured surfaces, or hybrid organic–inorganic matrices. Adjunctive biologics such as PRF or BMP-2 markedly influence healing dynamics, indicating that future regenerative strategies may rely on synergistic combinations of scaffold design and biologic stimulation rather than on scaffold characteristics alone.

However, variability in preparation protocols, reproducibility, and lack of standardization represent key limitations when interpreting studies on biologically enriched approaches—particularly PRF and albumin-coated grafts—in maxillary sinus augmentation. With regard to PRF, substantial heterogeneity exists across studies in terms of centrifugation devices, relative centrifugal force, centrifugation time, tube material, and PRF subtype (e.g., L-PRF, A-PRF, injectable PRF). These variables significantly influence fibrin architecture, cellular composition, and growth factor release kinetics, ultimately affecting biological performance [57]. In addition, patient-related factors such as age, hematological profile, and systemic conditions further contribute to inter- and intra-study variability. Importantly, many publications do not report these parameters in sufficient detail, limiting reproducibility and comparability of outcomes. Similar concerns apply to albumin-coated grafts and albumin-enriched PRF formulations, which represent relatively novel approaches. Currently, there is no consensus regarding albumin source, processing method (e.g., thermal denaturation parameters), graft–albumin ratios, or handling protocols. As a result, the available evidence is derived from small, heterogeneous cohorts using center-specific preparation techniques, making cross-study comparisons and external validation challenging [58].

## 6. Future Perspectives and Directions

More advanced in vitro approaches, including co-culture systems, three-dimensional models, and dynamic culture conditions, may better mimic the vascularized and multicellular nature of the maxillary sinus environment [59]. The integration of osteogenic, endothelial, and immune cell populations could improve the translational relevance of experimental findings by more accurately reflecting the processes of angiogenesis, osteogenesis, and biomaterial–tissue interactions observed in vivo [60].

In preclinical investigations using animal models, anatomical and physiological differences among commonly employed species—such as rabbits, dogs, sheep, and minipigs—affect sinus morphology, bone turnover rates, vascularization patterns, and membrane thickness, all of which can influence graft integration and bone regeneration dynamics. Consequently, outcomes observed in one species may not be directly extrapolated to another or to the human maxillary sinus. Moreover, defect size significantly impacts regenerative results, as smaller or non–critical-size defects may heal spontaneously, potentially leading to an overestimation of the osteogenic potential of a biomaterial, whereas larger or critical-size defects provide a more stringent and clinically relevant evaluation. Healing timelines also strongly influence reported outcomes: early time points primarily reflect inflammatory responses and initial osteoconductive processes, while longer observation periods are necessary to assess graft resorption, bone maturation, and remodeling. Therefore, future research should carefully consider these variables, and conclusions regarding biomaterial performance should be interpreted within the context of the specific animal model, defect configuration, and follow-up duration employed.

Although several synthetic and composite grafts frequently demonstrated enhanced early vascularization and accelerated bone formation, current clinical evidence does not conclusively indicate that these early advantages persist over longer healing periods. In studies with extended follow-up, initial differences between synthetic grafts and xenografts often attenuate as bone remodeling progresses, with outcomes converging in terms of mature bone volume and quality. Importantly, implant survival and success rates reported to date appear broadly comparable across different grafting materials when appropriate surgical protocols and healing times are respected. In this context, bovine-derived xenografts—particularly Bio-Oss^®^—have consistently shown favorable long-term clinical outcomes, including stable graft integration and high implant survival rates in regenerative procedures, as supported by recent systematic evidence [61]. Nevertheless, the available clinical literature is characterized by substantial heterogeneity in study design, follow-up duration, and outcome assessment, which limits direct long-term comparisons among graft materials. Consequently, it remains unclear whether the accelerated early osteogenesis observed with some synthetic grafts translates into a measurable long-term advantage in implant survival or stability. This point remains insufficiently clarified in the current literature and should be addressed in future research.

Moreover, despite the proven strong osteoinduction, growth-factor-enhanced materials like BMP-2 face significant barriers to widespread clinical translation, primarily related to safety concerns at high doses, challenges in achieving controlled delivery, stringent regulatory pathways, and substantial costs [62]. The primary safety concern stems from the high doses often required with conventional delivery systems to achieve efficacy due to the short half-life of the protein, which can lead to supraphysiological levels locally and systemically [63]. Achieving a balance between efficacy and safety requires precise dose control, which is currently a major challenge in future research. The path to regulatory approval for growth factor products is complex, largely due to the challenges in demonstrating consistent safety and efficacy. Furthermore, the substantial economic burden associated with growth factor technologies represents a major barrier to their accessibility and widespread clinical implementation.

In addition, we have emphasized that the biological and clinical effects of PRF- and albumin-based approaches appear to be highly protocol-dependent; thus, the need for standardized preparation protocols with clearly defined and reported parameters emerges. Until such standardization is achieved, the interpretation of outcomes and generalization of results across studies should be approached with caution.

Finally, advancements in imaging, defect modeling, and delivery systems show that procedural innovation is evolving in parallel with material development.

## 7. Conclusions

In conclusion, the integration of in vitro, in vivo, and clinical evidence depicts a field progressing toward biomaterials that are bioactive, resorbable, supportive of vascularization, and increasingly customizable—materials capable not only of preserving sinus volume but also of actively promoting the biological mechanisms underpinning bone regeneration. Rather than indicating convergence on a single superior graft, current data suggest that optimal outcomes emerge from the interplay between advanced scaffold design, biologic enhancement, and optimized clinical protocols. This multidimensional strategy represents the future trajectory of sinus augmentation and holds substantial potential for reducing healing times, improving bone quality, and increasing the predictability of implant rehabilitation in the posterior maxilla.

## Figures and Tables

**Table 1:** Biomaterial innovation in animal models; for each study, the biomaterial under investigation, the animal model, the evaluation timeline, and the key findings have been highlighted.

Group	Biomaterial	Animal Model & Timing	Evaluation Method	Key Findings	Study Ref.
DBBM	DBBM (small vs. large granules)	Rabbit 2, 4, 8 weeks	Histology; histomorphometric analysis of sinus membrane thickness and perforations; light microscopy.	Progressive Schneiderian membrane thinning and perforations in both particle sizes; effect intrinsic to DBBM.	[21]
	Collagenated DBBM vs. DBBM	Rabbit 2 and 12 weeks	Histology; histomorphometry of membrane integrity and new bone formation at early and late time points.	Early membrane protection with collagen, but by 12 weeks, both materials show similar thinning and perforation; similar bone formation.	[22]
	DBBM + hyaluronic acid + polynucleotides	Rabbit 2 and 10 weeks	Histology; histomorphometric quantification of new bone, residual graft, and membrane alterations.	No improvement in bone formation or membrane condition; adjunct gel does not enhance DBBM performance.	[23]
	Autograft vs. DBBM substitute	Mouse Days 0, 9, 18, 120	Histology; alkaline phosphatase (ALP) activity assays; histomorphometry at multiple time points; long-term tissue evaluation.	Autograft shows fast early osteogenesis; DBBM slower initially but supports later bone formation; DBBM remains slowly resorbing long-term.	[24]
Porcine xenografts	Sticky porcine graft vs. particulate porcine	Rabbit 8–10 weeks	Micro-CT for volume stability; histology; histomorphometry of new bone and graft displacement.	Sticky graft reduces particle extrusion; both show ~50% volume loss; similar bone regeneration.	[25]
	Collagenated porcine xenograft vs. DBBM	Rabbit 2–8 weeks	Histology; histomorphometry of bone formation and graft resorption; sinus wall bone contact analysis.	Faster resorption and greater shrinkage with porcine collagen graft; increased bone along sinus walls compared to DBBM	[26]
	Particulate porcine vs. collagenated porcine block	Beagle dog 8 weeks	Histology; histomorphometry of new bone, residual graft, and osseointegration at 8 weeks.	Block graft shortens insertion time; new bone, graft remnants, and osseointegration similar between groups.	[27]
Synthetic composites	PLGA scaffold	Sheep 16–28 weeks	Micro-CT; histological analysis; histomorphometric evaluation of bone remodeling and scaffold integration.	Good biocompatibility, progressive bone remodeling and integration; promising long-term maturation pattern	[28]
	Biosilicate^®^ vitroceramic	Rabbit 15, 45, 90 days	Histology; inflammatory cell scoring; histomorphometric analysis of bone and granulation tissue over time.	Adequate bone formation but persistent inflammation and granulation tissue; biocompatibility concerns for sinus use.	[30]
	Gelatin/nanoclay colloidal gel (injectable, self-healing)	Rat/Mouse	Histology; histomorphometry; immunohistochemical markers of osteogenesis; in vivo bone ingrowth assessment.	Supports cell adhesion, osteogenic differentiation, bone ingrowth; injectable and conformable; favorable resorption; promising for minimally invasive grafting.	[31]
	Porous titanium granules	Sheep 30, 60, 90 days	Micro-CT; histology; histomorphometric evaluation of osteoconduction and bone–material contact.	Strong osteoconduction and high bone formation, but remains permanently in place; non-resorbable long-term.	[32]
Bioactive/growth-factor enhanced	rhBMP-2 + absorbable collagen sponge; vs. addition of biphasic ceramic	Mini-pig Several weeks	Micro-CT; histology; histomorphometry of newly formed bone; comparative quantitative analysis.	BMP-2 drives strong osteoinduction regardless of scaffold type; ceramic addition provides no additional benefit.	[34]
	Heparin-modified gelatin nanofiber gel + BMP-2	Rat 8 weeks	Micro-CT; histology; quantitative bone volume assessment; osteogenesis scoring.	Highest osteogenic response among compared materials; potent early bone formation with controlled BMP-2 release	[35]

**Table 2:** Clinical cases: biomaterial, project aim and the evaluation methods adopted, main findings have been highlighted.

Biomaterial	Project Aim and Evaluation Methods	Main Findings	Study Ref.
Octacalcium phosphate (OCP)–based synthetic graft: Bontree^®^	Develop a fully synthetic, resorbable, bioactive alternative to xenografts. Histological analysis; histomorphometry; immunohistochemical markers of osteogenesis (ALP activity, RANKL expression); clinical implant follow-up.	Enhanced osteoblastic activity, reduced osteoclastogenesis; successful human graft integration; promising biologically active synthetic substitute.	[36]
Mg-hydroxyapatite/collagen scaffold (MgHA–collagen)	Create a bioinspired hybrid scaffold mimicking natural bone and remodeling faster than xenografts. Cone Beam CT; histology; scanning electron microscopy; histomorphometric quantification at 6 months.	At 6 months, graft largely replaced by new bone; high cellularity and vascularization; no residual scaffold; stable regenerated volume.	[37]
PRF + DBBM or autograft	Develop imaging-based (non-radiation) monitoring and test PRF-induced angiogenesis. Contrast-enhanced magnetic resonance imaging (MRI); long-term radiological follow-up; qualitative and quantitative assessment of graft vascularization and remodeling.	PRF accelerates vascularization and bone maturation; MRI effective for long-term graft assessment; PRF side shows better remodeling at 10 years.	[38]
PRF vs. Allograft	To evaluate whether PRF alone can serve as a grafting material for direct sinus lift, reducing the need for bone allografts. The residual bone height (RBH), implant mobility, and soft tissue characteristics between and within the groups were compared and analyzed.	Both groups gained bone height, but PRF alone produced significantly greater RBH increase at 6–9 months; demonstrated predictable and stable sinus augmentation without use of graft materials.	[39]
A-PRF + serum albumin–coated allograft (SACBA)	Assess biological enhancement to accelerate healing. Histomorphometry; micro-CT–based trabecular microarchitecture analysis comparing 3- and 6-month healing periods.	Similar bone quality and volume at 3 and 6 months; suggests healing time can be reduced using A-PRF + SACBA combination.	[40]
Six biomaterials: MCBA–Mineralized Cancellous Bone Allograft; FDBA–Freeze-Dried Bone Allograft; ABB (Bio-Oss^®^)–Anorganic Bovine Bone; EB–Equine Bone; BCP–Biphasic Calcium Phosphate; Bioapatite–collagen composite	Compare performance of allografts, xenografts, and synthetics under identical conditions. Histological analysis of bone biopsies; (new bone, residual graft, connective tissue fractions).	All biocompatible; FDBA showed highest new bone (32%); ABB lowest (~16%); modern allografts may outperform xenografts in early formation.	[41]
Bio-Oss vs. Cerabone (bovine xenografts)	Bio-Oss vs. Cerabone (bovine xenografts) Micro-CT analysis was performed to assess bone volume fraction, trabecular thickness, number, and separation.	Both effective, but Cerabone produced significantly more new bone and higher bone volume fraction at 6 months than Bio-Oss, indicating superior regenerative performance.	[42]
Bio-Oss ± collagen membrane over lateral window	Test whether membrane on osteotomy window improves bone yield. Histomorphometric analysis of biopsies; micro-CT analysis of regenerated bone volume and architecture.	No difference in bone quantity or quality; membrane coverage of lateral window may be unnecessary.	[43]
Biphasic calcium phosphate (BCP) graft (30% HA/70% β-TCP)	Track temporal regeneration. Histology; Raman spectroscopy; macromolecular composition analysis; temporal assessment of mineral and collagen maturation.	Progressive remodeling at 6–12 months; β-TCP component resorbs steadily; increasing collagen and mineral organization; BCP acts as a dynamic scaffold.	[44]
β-TCP macro/micro-porous 3D-printed scaffold (Lithography-based Ceramic Manufacturing (LCM))	To create a fully synthetic, architecturally optimized scaffold for sinus augmentation and jawbone regeneration. Postoperative evaluation: µCT, synchrotron imaging, histology, and Fourier Transform Infrared Imaging.	Highly porous, mechanically stable lattice supporting active new bone formation and controlled scaffold resorption in human biopsy; confirmed good osteoconductivity and biocompatibility.	[45]
Collagenated porcine bone (mp3^®^)	Evaluate materials with preserved natural collagen for better clot stability and cell migration. Synchrotron radiation X-ray microtomography; histological evaluation; 3D quantitative trabecular analysis over time.	Gradual graft resorption; excellent trabecular organization; improved early integration and cellular infiltration.	[46]

**Table 3:** A schematic overview of the currently most used polymers and composite materials for sinus augmentation.

	Observation	Interpretative Meaning
Most frequently used polymer	Collagen (animal and human studies)	Collagen is the dominant polymer due to its biocompatibility, biological signaling capacity, and regulatory acceptance.
Roles of collagen	Structural matrix, biological carrier, barrier membrane	Collagen serves multifunctional roles: scaffold integration, growth-factor delivery, clot stabilization, and soft-tissue protection.
Synthetic polymers	PLGA used exclusively in animal models	Synthetic polymers are still in an exploratory or preclinical phase, likely due to regulatory and degradation predictability concerns.
Injectable polymeric systems	Gelatin, fibrin (PRF), polymeric gels	Injectable polymers improve handling, reduce invasiveness, and better adapt to sinus anatomy, favoring early healing.
Polymer–ceramic composites	Collagen–HA, gelatin–β-TCP, polymer–xenograft hybrids	Combining polymers with ceramics balances mechanical stability with biological activity and controlled resorption.
Purely ceramic vs. polymer-containing grafts	Ceramics dominate routine clinical use; polymers more common in experimental studies	Polymer-based systems are being tested to overcome the biological passivity of ceramics, but clinical adoption remains cautious.
Biological function of polymers	Angiogenesis support, cell recruitment, controlled degradation	Polymers act as biological modulators rather than load-bearing elements in sinus augmentation.

## Data Availability

No new data were created or analyzed in this study. Data sharing is not applicable to this article.

## Abbreviations

The following abbreviations are used in this manuscript:

DBBM	deproteinized bovine bone mineral
β-TCP	β-tricalcium phosphate
HA/	hydroxyapatite/
β-TCP	β-tricalcium phosphate
PLGA	poly(lactic-co-glycolic acid)
BMP-2	bone morphogenetic protein-2
OCP	octacalcium phosphate
PRF	platelet-rich fibrin
BCP	biphasic calcium phosphate
LCM	lithography-based ceramic manufacturing
Micro-CT	X-ray micro computed tomography
FTIR	Fourier-transform infrared
